# Macrolide-Resistant and Macrolide-Sensitive *Mycoplasma pneumoniae* Pneumonia in Children Treated Using Early Corticosteroids

**DOI:** 10.3390/jcm10061309

**Published:** 2021-03-22

**Authors:** Hye Young Han, Ki Cheol Park, Eun-Ae Yang, Kyung-Yil Lee

**Affiliations:** 1 Department of Pediatrics, College of Medicine, The Catholic University of Korea, 222, Banpo-daero, Seocho-gu, Seoul 06591, Korea; unaver@hanmail.net (H.Y.H.); leekyungyil@catholic.ac.kr (K.-Y.L.); 2 Department of Pediatrics, Daejeon St. Mary’s Hospital, The Catholic University of Korea, 64 Daeheung-ro, Jung-gu, Daejeon 34943, Korea; 3 Clinical Research Institute, Daejeon St. Mary’s Hospital, The Catholic University of Korea, Daejeon 34943, Korea; kcpark@cmcnu.or.kr; 4 Junglock Biomedical Institute, 127, Yuchun-ro, Jung-gu, Deajeon 34886, Korea

**Keywords:** *Mycoplasma pneumoniae*, macrolide, pneumonia, corticosteroid, child

## Abstract

We have found that early corticosteroid therapy was effective for reducing morbidity during five Korea-wide epidemics. We evaluated the clinical and laboratory parameters of 56 children who received early corticosteroid treatment for pneumonia that was caused by macrolide-resistant *Mycoplasma pneumoniae* (*M. pneumoniae*) or macrolide-sensitive *M. pneumoniae* between July 2019 and February 2020. All subjects had dual positive results from a PCR assay and serological test, and received corticosteroids within 24–36 h after admission. Point mutation of residues 2063, 2064, and 2067 was identified in domain V of 23S rRNA. The mean age was 6.8 years and the male:female ratio was 1.2:1 (31:25 patients). Most of the subjects had macrolide-resistant *M. pneumoniae* (73%), and all mutated strains had the A2063G transition. No significant differences in clinical and laboratory parameters were observed between macrolide-resistant and macrolide-sensitive *M. pneumoniae* groups that were treated with early dose-adjusted corticosteroids. Higher-dose steroid treatment may be needed for patients who have fever that persists for >48 h or increased biomarkers such as lactate dehydrogenase concentration at follow-up despite a usual dose of steroid therapy.

## 1. Introduction

*Mycoplasma pneumoniae* (*M. pneumoniae*) is a common cause of community-acquired pneumonia in children and young adults [1]. Epidemics of *M. pneumoniae* pneumonia have occurred in 3–4-year cycles in South Korea, with the most recent epidemic occurring in 2019 [2,3]. Although most patients with *M. pneumoniae* pneumonia have mild symptoms and a self-limited clinical course, some patients experience severe or refractory pneumonia and/or extrapulmonary complications, such as encephalopathy, Stevens-Jonson syndrome, small-vessel cutaneous vasculitis, myositis, and acute kidney injury, as well as other organ involvement [4,5,6].

Because *M. pneumoniae* does not have a cell wall, it is resistant to beta-lactams, fosfomycin, and glycopeptide antibiotics. However, *M. pneumoniae* is very sensitive to some antibiotics in vitro, such as macrolides, fluoroquinolones, and tetracyclines, which are used to treat *M. pneumoniae* infection. Macrolides are used as first-line antibiotics for children because they have a lower adverse drug reaction, although macrolide-resistant *M. pneumoniae* strains have recently become prevalent in East Asian countries, including Japan, China, and South Korea [7,8,9]. In Korea, the majority of *M. pneumoniae* infections showed macrolide-resistant *M. pneumoniae* strains with residue 2063 mutation in domain V of 23S rRNA, and macrolide-resistant *M. pneumoniae* infection may also be increasingly common in other countries [9,10]. Thus, new treatment strategies have emerged for patients with macrolide-resistant *M. pneumoniae* strains in Korea and other countries [11].

Some groups have reported that patients with macrolide-resistant *M. pneumoniae* pneumonia have more severe clinical manifestations, such as longer fever duration and hospitalization, relative to patients with macrolide-sensitive *M. pneumoniae* pneumonia [7,10,12]. However, other groups have reported that macrolide-resistant *M. pneumoniae* pneumonia might respond to macrolide treatment and that some clinical indices did not differ between macrolide-resistant and macrolide-sensitive *M. pneumoniae* pneumonia [13,14]. These findings suggest that other treatment modalities may be needed for antibiotic-unresponsive or severe *M. pneumoniae* pneumonia. Although the effects of antibiotic treatment on *M. pneumoniae* infection in children remain controversial, pediatricians have used alternative antibiotics and/or additional immune modulators, such as corticosteroids, for antibiotic-nonresponsive or refractory macrolide-resistant *M. pneumoniae* pneumonia. We have also used corticosteroids for treating patients with severe *M. pneumoniae* pneumonia since the 2003 epidemic in Korea and have reported that early corticosteroid treatment was effective for reducing morbidity through five recent nationwide epidemics [15,16,17]. Therefore, this study aimed to evaluate the clinical and laboratory parameters of children who received early corticosteroid treatment for pneumonia caused by macrolide-resistant or macrolide-sensitive *M. pneumoniae.*

## 2. Materials and Methods

### 2.1. Ethical Considerations

The study protocol was approved by the institutional review board of the Daejeon St Mary’s Hospital, The Catholic University of Korea (DC20SASI0075).

### 2.2. Subjects

This study evaluated previously healthy children who were diagnosed with *M. pneumoniae* pneumonia and admitted to The Catholic University of Korea Daejeon St. Mary’s Hospital between July 2019 and February 2020. All eligible patients had positive *M. pneumoniae* results from a PCR assay and serological test. The exclusion criteria were patients with a negative *M. pneumoniae* result from the PCR assay and patients with a positive PCR result but a negative serological result.

The serological test was performed using a commercial ELISA kit (Diesse Diagnostica, Senese, Italy), and the IgM results were categorized according to the manufacturer’s instructions as positive (>1.1), negative (<0.9), or equivocal (0.9–1.1). A case of *M. pneumoniae* pneumonia was identified based on a positive IgM result or seroconversion (negative or equivocal to positive) in a child with pneumonia through 2 times serologic tests during hospitalization. Nasopharyngeal aspirate samples from 56 patients had been subjected to a multiplex bacterial PCR assay, which included *M. pneumoniae*, using the CFX96 Touch TM Real-Time PCR detection system (Bio-Rad, Hercules, CA, USA). The remaining PCR materials were also stored for genetic analysis.

All patients had been treated using corticosteroids within 24–36 h after admission. The initial treatment in cases with milder pneumonia lesions involved oral prednisolone (1 mg/kg/day) or intravenous methylprednisolone (1–2 mg/kg/day), although a higher dose (5–10 mg/kg/day) was used for patients with more severe segmental/lobar lesions or respiratory distress (e.g., wheezing or tachypnea at presentation). If a patient exhibited persistent fever and disease progression at 48–72 h after the initial steroid therapy, an additional high-dose steroid was used (10–30 mg/kg/day) based on laboratory values and clinical severity. Pulmonary infiltration was evaluated by two authors (E.-A.Y. and H.Y.H.) based on the chest radiograph findings, which were classified as bronchopneumonia or segmental/lobar pneumonia. Medical records were reviewed to collect data regarding clinic-demographic characteristics, chest radiograph findings, and laboratory findings.

### 2.3. Nucleic Acid Extraction and PCR Analysis

All specimens containing *M. pneumoniae* DNA had been stored at −70 °C before testing. Nucleic acid extraction was performed using the Ribospin TM vRD plus kit (GeneAll Biotechnology, Seoul, Korea) according to the manufacturer’s instructions. The extracted DNA was subjected to PCR using the Pfu Plus 5× Master mix (Elpis Biotech, Daejeon, Korea) according to the manufacturer’s instructions. The primers targeted residues 1998–2018 (forward) and 2673–2692 (reverse) in domain V of 23S rRNA (Myco23S-F: 5-TCTCGGCTATAGACTCGGTGA-3 and Myco23S-R: 5-TAAGAGGTGTCCTCGCTTCG-3).

The PCR products were subjected to 1% agar gel electrophoresis using a submarine-type electrophoresis device (Mupid-exU; Takara Bio Inc., Shiga, Japan). The approximately 700-bp band was identified under ultraviolet light, photographed using an imaging system (Geldoc XR image system; BioRad, Hercules, CA, USA), and then excised. The gel extraction procedure was performed using a commercial gel extraction SV kit (MGmed, Seoul, Korea), and the product was analyzed using the BigDye^®^ Terminator v3.1 Cycle Sequencing and an ABI PRISM 3730XL Analyzer (Applied Biosystems, Thermo Fisher Scientific, Foster City, CA, USA). The DNA sequences were evaluated for point mutations at residues 2063, 2064, and 2067 in domain V of 23S rRNA using a sequence that is registered in the GenBank database (GenBank number: X68422).

### 2.4. Statistical Analysis

All analyses were performed using SPSS software (version 14.0; SPSS Inc., Chicago, IL, USA). Continuous data were reported as median (interquartile range (IQR)), while categorical data were reported as number (percentage). Inter-group comparisons were performed using the Mann–Whitney test for continuous variables and the chi-squared test or Fisher’s exact test for categorical variables. Correlations between two continuous variables were analyzed using Pearson’s correlation coefficient. All *p*-values were two-tailed, and differences were considered statistically significant at *p*-values of <0.05.

## 3. Results

The study included 56 patients with positive results from the PCR assay and serological test. The mean age was 6.8 years (min-max: 1–15 years), and the male: female ratio was 1.2:1 (31:25). Most patients (41/56 patients, 73.2%) had *M. pneumoniae* with the A2063G mutation (the macrolide-resistant *M. pneumoniae* group), although 15 patients (the macrolide-sensitive *M. pneumoniae* group) had *M. pneumoniae* with no mutations at residues 2063, 2064, or 2067. There were no significant differences between the macrolide-resistant or macrolide-sensitive *M. pneumoniae* groups in terms of age, sex, fever duration before and after admission, total fever duration, admission duration, and chest radiograph findings (Table 1). The serological test results revealed IgM positivity at admission for 36 patients and IgM negativity at admission for 20 patients, although those patients had experienced seroconversion by the second examination. There were no significant inter-group differences in the serological test results (Table 1).

All patients initially received corticosteroid treatment within 24–36 h after admission. Most patients (46/56 patients, 82%) received low-dose corticosteroids (oral prednisolone at 1 mg/kg/day or intravenous methylprednisolone at 1–2 mg/kg/day), although 10 patients with more severe symptoms received high-dose steroid (5–10 mg/kg/day). After the treatment, 42 patients (75%) experienced defervescence within 24 h, 53 patients (94.6%) experienced defervescence within 48 h, and 54 patients (96.4%) experienced defervescence within 72 h. There was no significant difference in defervescence between the two groups. However, 2 patients who were infected with macrolide-resistant *M. pneumoniae* received additional antibiotics (levofloxacin) and corticosteroids (20 and 30 mg/kg/day, respectively) due to persistent fever and rapid disease progression, based on deterioration of the chest radiographic findings and progressive pleural effusion at 48 h after starting initial therapy. Both completely improved without long-term complications. None of the patients received alternative antibiotics during their clinical course except for these two patients. There were no significant differences between the two groups in the corticosteroid treatments (Table 1).

Laboratory tests were performed for all patients at admission. No significant differences were observed between the macrolide-resistant and macrolide-sensitive *M. pneumoniae* groups in any of the laboratory markers. Of these, 33 were followed up on the 3rd or 4th day of hospitalization. At initial and follow up tests, laboratory markers including white blood cell count (WBC) and differential, aspartate aminotransferase (AST), alanine aminotransferase (ALT), alkaline phosphatase (ALP), lactate dehydrogenase (LDH), erythrocyte sedimentation rate (ESR), and C-reactive protein (CRP) showed statistically significant differences (Table 2).

## 4. Discussion

The present study revealed that 73% of the subjects had the A2063G mutation in domain V of 23S rRNA during the 2019 *M. pneumoniae* epidemic in Korea. One of macrolides, erythromycin, has a 14-membered structure that inhibits protein synthesis by interacting with the peptidyl transferase loop of the 23S rRNA. Mutations at residues 2063, 2064, 2067, and 2617 are associated with macrolide resistance [18], although the A2063G transition in the central loop is reportedly the major source of macrolide resistance in *M. pneumoniae*, as mutations at residues 2067 and 2617 are rare [19]. In Korea, approximately 60–87% of children have the A2063G mutant [10,13,20]. The prevalence of macrolide-resistant *M. pneumoniae* was reportedly 87% in Japan during 2011 [21], although other Japanese groups had reported that the prevalence of macrolide-resistant *M. pneumoniae* has decreased recently in Japan [22]. In contrast, Korea and China have reported high proportions of macrolide-resistant *M. pneumoniae* infections until recently [12,23]. A lower prevalence has been reported in the US (mean: 7.5%, range: 1.9–21.7%) [14], and an even lower prevalence has been observed in some European countries [24,25]. Thus, the proportion of macrolide-resistant *M. pneumoniae* likely varies according to region as well as to time period and the inherent epidemiological situation.

Previous studies have demonstrated that macrolide-resistant *M. pneumoniae* can prolong the durations of fever and hospital stay and is associated with a higher frequency of alternative antibiotic treatment after macrolide treatment, relative to macrolide-resistant *M. pneumoniae* [7,8,12,19,26]. Meanwhile, some studies have failed to detect significant differences in clinical presentation, radiographic findings, complications, or admission duration [13,14,20]. The present study also failed to detect significant differences between the macrolide-resistant or macrolide-sensitive *M. pneumoniae* groups in terms of clinical and laboratory parameters at presentation, including IgM positivity and corticosteroid responsiveness. These results may be influenced by early corticosteroid use, as most patients in both groups experienced defervescence within 3 days.

The pathogenesis of lung injury during *M. pneumoniae* infection and other infectious diseases such as COVID-19 remains unclear. The host’s hyperactive immune response against the infection’s insults may be related to host cell injury [1,27]. The host immune system may respond to substances produced by these pathogens (e.g., toxins and pathogen-associated molecular patterns), as well as to substances produced by infected host cells, such as pathogenic proteins, peptides, and damage-associated molecular patterns. In cases of pneumonia, substances produced by injured lung cells can induce even greater inflammation if they are released into the systemic circulation or local environment. Therefore, it is crucial to control the initial hyperactive immune response in order to reduce morbidity and prevent pneumonia progression. Given the dose-dependent effects of corticosteroids, higher doses may be needed for patients with severe pneumonia to help control the effects of pathogenic substances and the corresponding immune responses during the acute phase [28,29]. We have also previously reported that antibiotics may only have a limited effect on *M. pneumoniae* infection [17]. For these reasons and our experiences, we use steroids to treat *M. pneumoniae* pneumonia, but there has been an ongoing debate about the initiation time and dose of steroid therapy for *M. pneumoniae* infection [16,17,19].

There is no clear definition for severe antibiotic non-responsive or refractory *M. pneumoniae* pneumonia, although some studies have defined these cases as having a prolonged fever and lung lesion progression, despite 7 days of appropriate antibiotic treatment. We have encountered a few patients who did not respond to initial corticosteroid treatment and required additional corticosteroids during the 2015–2016 epidemic and in the present study [11,17]. Several studies have attempted to identify biomarkers that can predict refractory pneumonia. For example, potentially useful biomarkers for severe or refractory pneumonia include AST, ALT, LDH, CRP, ferritin, and various cytokines (IL-18, IL-6, or TNF-α) [4,30,31,32]. Furthermore, the initial immunological insults can induce further inflammation and possibly secondary bacterial invasion, which suggests that elevated values for LDH, CRP, and some biomarkers may reflect the degree of lung tissue injury and an increased risk of other organ involvement. Therefore, follow-up evaluation of these markers may be useful for monitoring disease progression. In our study, two patients who received additional high-dose steroids had the highest initial LDH concentrations, which increased after 3 days of the initial steroid treatment. Thus, we decided to use additional higher dose corticosteroid (20 and 30 mg/kg/day). In contrast, most patients had lower LDH concentrations at the follow-up examination, relative to their initial value. Because early control of ongoing inflammation is important and some biomarkers reflect inflammation severity, more aggressive treatments (e.g., high-dose pulse steroid treatment) based on the follow-up laboratory values and clinical severity, such as the patient’s persisting fever >48 h after starting the low-dose steroid treatment, may be needed.

The present study has several limitations. First, the sample size was small and selection bias is possible. However, we attempted to ensure that all patients had *M. pneumoniae* pneumonia by only including patients with dual positive results from the PCR assay and serological test. Second, relative to previous studies that were conducted at tertiary hospitals, there was a larger number of patients with mild pneumonia and early presentation. Third, this study was not a comparative study for corticosteroid effect because there was no control group with this respect.

## 5. Conclusions

The present study revealed macrolide-resistant *M. pneumoniae* in 73% of the subjects during the 2019 epidemic in Korea, and all mutated strains had the A2063G mutation in domain V of 23S rRNA. There were no differences in clinical and laboratory parameters in either the macrolide-resistant or the macrolide-sensitive *M. pneumoniae* groups that were treated with early, dose-adjusted, corticosteroid therapy. In the era of macrolide-resistant *M. pneumoniae* strains, proper treatment modalities may be needed for severe antibiotic-unresponsive pneumonia patients.

## Figures and Tables

**Table 1:** Comparing the clinical characteristics of the macrolide-resistant or macrolide-sensitive *M. pneumoniae* groups.

Characteristic	All Patients	MRMP	MSMP	*p*-Value
	*n* = 56	(*n* = 41)	(*n* = 15)	
Age (years)	6.8 (5.0–9.1)	6.3(4.7–8.8)	7.0 (5.8–10.3)	0.264
Male:female ratio	31:25	25:16	6:9	0.162
Hospitalization (days)	5 (4–6)	5 (4–6)	5 (5–6)	0.774
Duration of fever (days)				
Before admission	5.0 (3.3–7.0)	5.0 (3.5–7.0)	5.0 (2–7.0)	0.519
Total duration	5.0 (3.3–7.0)	6.0 (3.5–7.5)	5.0 (3.0–7.0)	0.864
Pulmonary infiltration, *n* (%)				
Bronchopneumonia	16 (28.6)	12 (29.3)	4 (26.7)	0.849
Segmental/lobar pneumonia	40 (71.4)	29 (70.7)	11 (73.3)	
Serological test, *n* (%)				
Initial ELISA IgM positive	36 (64)	27 (66)	9 (60)	0.686
IgM seroconversion	20 (36)	14 (34)	6 (40)	
Antibiotics, *n* (%)				
Macrolide, *n* (%)	51(91)	38 (93)	13 (87)	0.484
Levofloxacin, *n* (%)	2 (4)	2 (5)	0	
Corticosteroids, *n* (%)				
Low-dose (1–2 mg/kg)	46 (82)	34 (83)	12 (80)	1.000
High-dose (5–10 mg/kg)	10 (18)	7 (17)	3 (20)	
Additional high-dose (10–30 mg/kg)	2	2	0	

MRMP: macrolide-resistant *Mycoplasma pneumoniae*; MSMP: macrolide-sensitive *Mycoplasma pneumoniae.* Continuous variables are expressed as median (interquartile range (IQR)), and categorical variables are expressed as case’s absolute number (percentage).

**Table 2:** Comparison of laboratory findings during admission.

	At Presentation		*p* *	All Patients		*p* ^†^
	MRMP (*n* = 41)	MSMP (*n* = 15)		Initial (*n* = 56)	Follow-Up (*n* = 33)	
Hb	12.3 (11.5–12.9)	12.4 (11.8–13.3)	0.774	12.4 (11.7–13.0)	12.5 (12.0–13.2)	0.068
WBC	7.3 (5.7–8.8)	7.6 (6.1–8.7)	0.76	7.5 (5.7–8.7)	9.8 (7.9–14.1)	<0.001
N (%)	65.9 (53.4–70.3)	62.0 (48.0–67.8)	0.379	64.8 (52.6–70.2)	74.6 (58.5–79.3)	0.026
L (%)	24.0 (19.1–37.0)	25.2 (20.5–38.3)	0.494	24.8 (20.0–37.3)	18.2 (11.3–30.3)	0.033
M (%)	8.1 (6.4–9.1)	8.4 (6.9–9.2)	0.465	8.1 (6.8–9.1)	7.7 (5.0–10.9)	0.823
AST	27 (23–33)	26 (21–30)	0.262	27 (23–32)	24 (20–29)	<0.001
ALT	12 (10–16)	12 (8–14)	0.546	12 (10–15)	19 (13–26)	<0.001
ALP	169 (153–207)	167 (141–180)	0.259	168 (150–202)	163 (126–186)	0.022
LDH	271 (247–330)	250 (220–273)	0.085	269 (243–317)	270 (231–308)	0.007
ESR	14 (8–24)	17 (9–29)	0.691	14 (9–24)	11 (4–17)	0.004
CRP	1.7 (1.0–2.6)	2.2 (0.8–3.8)	0.505	1.8 (0.9–2.8)	0.4 (0.1–0.9)	<0.001

Values are presented as median (IQR). Hb: hemoglobin (g/dL), WBC: white blood cell count (×10^3^/mm^3^), N: neutrophil, L: lymphocyte, M: monocyte, AST: aspartate aminotransferase (U/L), ALT: alanine aminotransferase (U/L), ALP: alkaline phosphatase (U/L), LDH: lactate dehydrogenase (U/L), ESR: erythrocyte sedimentation rate (mm/h), CRP: C-reactive protein (mg/dL), MRMP: macrolide-resistant *Mycoplasma pneumoniae*, MSMP: macrolide-sensitive *Mycoplasma pneumoniae*, *p*: *p*-value. * Statistical analysis was performed between MRMP and MSMP groups. *p*-values were obtained using nonparametric *t*-test (Mann–Whitney test) for continuous variables. ^†^ Laboratory findings at admission and before discharge were compared. *p*-values were obtained using nonparametric paired *t*-test (Wilcoxon) for continuous variables.

## Data Availability

The data presented in this study are available on request from the corresponding author. The data are not publicly available due to restrictions of privacy.
